# Assessing barriers to dietary practices and healthy eating of female university students in nutrition programs, Jeddah, Saudi Arabia: a cross-sectional study

**DOI:** 10.3389/fpubh.2026.1864854

**Published:** 2026-09-07

**Authors:** Manal M. S. Mansoury, Afnan H. Saaty

**Affiliations:** Department of Food and Nutrition, Faculty of Human Sciences and Design, King Abdulaziz University, Jeddah, Saudi Arabia

**Keywords:** barriers, dietary practice, eating behaviors, nutrition programs, university students

## Abstract

**Objective:**

University students often experience challenges in maintaining healthy eating behaviors despite possessing adequate nutrition knowledge. This study assessed nutrition knowledge, eating habits, dietary practices, food-label use, and perceived barriers to healthy eating among female nutrition students in Saudi Arabia.

**Methods:**

A cross-sectional study was conducted among 343 undergraduate and postgraduate female students enrolled in nutrition programs at King Abdulaziz University, Saudi Arabia. Data were collected using a self-administered questionnaire assessing nutrition knowledge, eating habits, dietary practices, food-label and calorie on menu use, and perceived barriers to healthy eating. Descriptive statistics, Pearson correlation analyses, and multiple linear regression models were performed.

**Results:**

Over 90% of students recognized the health benefits of a healthy diet; however, dietary behaviors were only moderately healthy. The most commonly reported barriers were fast food availability (67.3%), the high cost of healthy foods (63.0%), social media influences on food choices (63.6%), and limited time for food preparation (52.8%). Healthier eating habits, healthier dietary practices, and greater use of nutritional information were associated with lower perceived barriers to healthy eating, particularly student-related and food-related barriers. Healthier eating habits were independently predicted by dietary practices (*β* = 0.311, *p* < 0.001), age (*β* = 0.248, *p* < 0.001), and lower perceived student- and food-related barriers. Healthier dietary practices were independently predicted by food-label use (*β* = 0.412, *p* < 0.001), healthier eating habits (*β* = 0.259, *p* < 0.001), and marital status (*β* = 0.110, *p* = 0.031).

**Conclusion:**

Despite enrolling in nutrition programs, students demonstrated only moderately healthy dietary behaviors, highlighting a knowledge–behavior gap. Interventions targeting practical, environmental, and social barriers may help promote healthier eating behaviors among future nutrition professionals.

## Introduction

Dietary habits are the consistent eating patterns individuals develop and maintain over time, shaped by internal, external, and intentional factors (1). The diet quality of college students is a rising concern in the field of public health (2). College is a critical time where students are likely to demonstrate increasing stress, poor eating habits, and irregular meal such as skipping breakfast, eating less fruits and vegetables, and more energy-dense foods (3). Such habits have been proven to affect diet quality and health detrimentally.

University is a transitional phase that students experience, making their dietary habits complex and multifaceted (4). In Saudi Arabia, studies show that university students often exhibit unbalanced dietary patterns (5). A recent study has demonstrated that an average of 42% of university students reported unhealthy eating habits. In addition, students had irregular meals, skipped breakfast, ate fast food frequently, and consumed insufficient amounts of vegetables, fruits, and water (6).

University students tend to face several barriers that make it difficult to eat healthy during the academic year, including lack of time, stress, financial constraints, insufficient availability of healthy foods, insufficient cooking skills, and social influences that normalize fast food and snacking (7, 8).

The rapid proliferation of Online Food Delivery (OFD) services during and in the wake of the COVID-19 pandemic is evident, particularly among the university student demographic that constitutes one of the most prevalent barriers toward their healthy eating (9, 10). Delivery apps in Saudi Arabia are now increasingly popular, allowing access to these services at any time (11). University student relay mostly on OFD, likely due to the combination of convenience, speed, and lack of available time to prepare meals (12).

Furthermore, many students tend to eat out frequently, influenced by social factors and the convenience of dining at restaurants, which often leads to poor food choices (13). The presence of calorie labelling in restaurants has been shown to guide healthier decisions as indicated by the Saudi Food and Drug Authority, but despite this, students often neglect the nutritional information and continue to make unhealthy choices (14). Similarly, Al-Barqi et al. revealed that only 27.4% of the university students stated that they always or usually use food labels when purchasing food products (15).

Social media platforms also play a significant role in shaping students’ dietary habits by promoting idealized body images, fast food, and unhealthy behaviors, contributing to body dissatisfaction and poor eating choices (16). Although calorie labelling and information on social media have the potential to encourage healthier eating, many students still face challenges in adhering to a balanced diet, influenced by convenience, peer behaviors, and the overwhelming amount of unhealthy food marketing.

One key factor influencing dietary habits is individuals’ knowledge and understanding regarding food and nutrition among university students. University students enrolled in programs related to health and food, such as nutrition and dietetics, are expected to demonstrate healthier eating habits compared to their peers due to the nature of their coursework (17). However, despite this knowledge, several studies have found that their eating behaviors do not always align with recommended healthy eating practices (18). This discrepancy raises the question of whether possessing nutrition-related knowledge is sufficient to translate into healthier dietary and lifestyle choices.

Despite the growing body of evidence on dietary behaviors among Saudi university students, to our knowledge, no research has comprehensively assessed the range of barriers faced specifically by students in nutrition programs. Most prior studies have targeted general student populations or focused on single dietary behaviors, without providing a multi-dimensional characterization of the barriers that impede healthy eating in this nutritionally literate group. Therefore, this study aimed to assess dietary practices and identify barriers to healthy eating among female university students enrolled in nutrition programs at King Abdulaziz University (KAU), Jeddah, Saudi Arabia. The findings are intended to contribute to the limited evidence available for this population and to inform future targeted interventions and multi-center studies across Saudi universities. Focusing specifically on female nutrition students is warranted for several converging reasons. First, from a professional standpoint, dietetics and nutrition is a predominantly female field globally and in Saudi Arabia. A study of dietitians practicing in Saudi hospitals found that 74.4% of participants were female (19). Similarly, a recently published research among dietitians in Saudi Arabia revealed that most respondents were female dietitians (83.57%) (20). Future female dietitians are therefore the primary workforce responsible for shaping the nutritional health of patients and communities, making it critical to understand and address dietary barriers they themselves may face during training. Moreover, from an institutional standpoint, KAU’s nutrition program serves a predominantly female student body, making this population a representative and accessible sample for understanding the lived dietary experiences of future nutrition professionals in the Kingdom.

## Subjects and methods

### Study design and sampling

This cross-sectional study was conducted in KAU, Jeddah, Saudi Arabia, from September to December 2025. An online self-administered questionnaire was used to collect data. The study population comprised female undergraduate (BSc) and Master (MSc) students from the Food and Nutrition Department, Faculty of Human Sciences and Design, and Clinical Nutrition Department, Faculty of Applied Medical Sciences. The inclusion criteria were female enrollment in different levels of the nutrition academic program and no health disorders or physiological conditions. The exclusion criteria include students who have any health condition that may affect their eating habits, have a diagnosis of an eating disorder, or follow a specific diet.

### Sample size and sampling technique

Sample size calculation was conducted using OpeEpi (21), a freely accessible epidemiological calculator. Assuming a total population of 513 female students enrolled in nutrition programs at King Abdulaziz University (KAU), an expected proportion of 50% (selected as a conservative estimate in the absence of prior data on the prevalence of healthy eating practices in this population), a margin of error of ±5%, and a 95% confidence level, the minimum required sample size was estimated to be 220 participants. To account for potential non-response, incomplete questionnaires, missing data, and invalid responses, the calculated sample size was increased by 20%. This adjustment is commonly recommended in cross-sectional survey research, particularly for self-administered questionnaires, to ensure adequate statistical power and representativeness despite potential data loss (22). Accordingly, the minimum required sample size was increased to 264 participants. A total of 343 students were ultimately included in the study, exceeding the minimum required sample size.

### Ethical approval

Ethical approval for this study was obtained from the Biomedical Ethics Research Committee, KAU, with ethical No. HA-02-J-008. Before participating, all participants received an informed consent form detailing the study’s purpose and their role in it. Participation was entirely voluntary and have the option to withdraw at any time without any repercussions. Additionally, all personal identifying information will remain confidential and anonymous.

### Study instrument and data collection process

Data was collected using an online questionnaire, which was distributed through the university’s official mailing list to reach all students in the nutrition academic program. The questionnaire was adapted from a previous study (23). Additionally, new questions have been added to assess social media usage, food ordering through delivery applications, and the use of food labels when purchasing food. These additional items were derived from previously published studies (11, 24, 25). The questionnaire contains five sections as follows:

Section 1—General information: This section contains some demographic and health-related factors data, including age, marital status, academic level, and the enrolled nutrition program. Participants were also asked to self-report their height (cm) and weight (kg) through the online questionnaire. These data were used to calculate body mass index (BMI), which was classified according to World Health Organization (WHO) criteria for BMI categories (26). Additionally, participants’ awareness of the concept of a healthy diet, alongside their knowledge of daily nutritional requirements.

Section 2—Eating habits: This section contains the frequency of their consumption of snacks, fried foods, fast foods, vegetables, fruits, and dates. Additionally, the frequency of dining outside the home, ordering meals from food delivery apps, consuming balanced meals, and sharing meals with friends and family. The response options for frequency include twice a day, once a day, 3–4 times a week, 1–2 times a week, and rarely. For healthy eating behaviors (e.g., consumption of fruits, vegetables, dates, balanced meals, and meal sharing), responses were scored from 1 to 5, with higher scores indicating more frequent healthy behaviors. For unhealthy eating behaviors (e.g., fried foods, fast foods, meals consumed outside the home, meals consumed at the university campus, and meals ordered from food delivery applications), reverse scoring was applied so that higher scores consistently reflected healthier eating habits. The total eating habits score was calculated by summing the responses across the 12 items, yielding a possible score range of 12–60, with higher scores indicating healthier eating habits.

Section 3—Dietary practices: This section assesses participants’ regularity in specific dietary practices including taking meals regularly, eating breakfast daily, eating breakfast outside home, including plant-based sources in main meals, considering the nutritional value of food, using iodized salt, and following a healthy diet. Items reflecting healthy dietary practices were scored from 1 to 4, with higher scores indicating greater adherence to healthy dietary practices. The item assessing breakfast consumption outside the home was reverse scored to ensure consistency in score direction. A total dietary practices score was obtained by summing responses across the seven items, resulting in a possible score range of 7–28, with higher scores indicating healthier dietary practices.

Section 4—Food labelling and calories on menus: This section examines participants’ engagement with food labels and calorie information, with questions on reading nutrient composition labels, considering the health effects of food products based on nutritional facts, regularly calculating daily calorie intake, looking at calories on restaurant menus when or before choosing an order, whether the number of calories displayed on menus influences their food choices, and whether they would consider buying lower-calorie foods if food delivery apps provided calorie information. Response options included always, sometimes, rarely, and never. Responses were scored on a four-point scale ranging from 1 to 4, with higher scores indicating greater engagement with food labelling and calorie information. The total food-labelling score was calculated by summing the six items, yielding a possible score range of 6–24, with higher scores reflecting greater utilization of nutritional information in food selection.

Section 5—Barriers to healthy eating: This section explores various factors that may hinder healthy eating including student-related factors, educational factors, food-related factors, environmental factors, social factors, social media use, ordering unhealthy food from delivery applications, and eating out. Participants responded to each statement using a three-point scale (agree, neutral, and disagree). Responses were scored from 1 to 3, with higher scores indicating greater disagreement with the barrier statement and therefore fewer perceived barriers to healthy eating. Scores were calculated separately for each barrier domain by summing the corresponding items. The possible score ranges were 5–15 for student-related barriers (5 items), 3–9 for educational barriers (3 items), 6–18 for food-related barriers (6 items), 2–6 for environmental barriers (2 items), 3–9 for social barriers (3 items), 4–12 for social media-related factors (4 items), 4–12 for barriers related to ordering unhealthy food through delivery applications (4 items), and 4–12 for barriers related to eating out (4 items). Higher scores reflected lower perceived barriers within each domain.

### Face validity and reliability testing

Face validity: To assess the face validity of the questionnaire, it was distributed to 5 experts for evaluation (27), which focused on three key aspects as outlined by Ehrenbrusthoff et al. (28), content completeness, comprehensibility, and time to complete. Experts were responding to the three questions below. It provided valuable insights into the overall usability of the questionnaire and helped identify any content or linguistic ambiguities, guiding further revisions if needed (29).

Completeness of content: “Do you believe this questionnaire covers the most important aspects related to the study’s objective? [Yes/No].” If “No,” they were asked to specify which aspects to add. All reviewers confirmed the completeness of the questionnaire’s content.

Comprehensibility: “Are the questions clear and easy to understand? [Yes/No].” If “No,” they were asked to identify which items are unclear. A limited number of comments were provided, which informed revisions to the questionnaire aimed at enhancing its clarity and comprehensibility.

Time to complete: “Is the time required to complete the questionnaire reasonable?” they rated this on a scale of 0–10, with 0 indicating “unacceptably long” and 10 meaning “completely acceptable.” The average obtained was 9.6, indicating the time needed to complete the questionnaire was reasonable.

Reliability testing: A pilot study including 69 participants was conducted to assess the internal consistency of the questionnaire. Data collected during the pilot phase were used solely for instrument refinement and were excluded from the final statistical analysis (*n* = 69), of the entire participants. The internal reliability of the questionnaire was measured using Cronbach’s alpha, which produced coefficients of 0.71 for the Eating Habits section, 0.72 for the Dietary Practice section, 0.78 for the Food Label Checking section, and 0.74 for the Barriers section. This shows that all measures/reliabilities of the study are at a respectable level (30). If any construct’s Cronbach’s *α* is below 0.7, specific questions may be revised or removed to enhance reliability. This process will ensure that the questionnaire demonstrates consistent performance and robustness for measuring the intended constructs.

### Statistical analysis

The collected data were computerized and statistically analysed using SPSS program version 27.0. Qualitative data were represented as frequencies and percentages. Quantitative data were assessed for normality using Kolmogorov–Smirnov (K–S) test and were expressed as mean ± SD. An independent *t*-test was used to calculate differences between quantitative variables.

Inferential analyses were conducted to examine relationships among dietary habits, dietary practices, food-labelling behaviors, and perceived barriers to healthy eating. Pearson correlation coefficients were calculated to assess bivariate associations between continuous variables. Subsequently, two multiple linear regression models were performed to identify independent predictors of eating habits and dietary practices, as key outcomes of the study. In the first model, the eating habits score was entered as the dependent variable, while age, marital status, BMI classification, level of study, dietary practices, food-labelling behaviors, and perceived barrier domains were included as predictors. In the second model, the dietary practices score was specified as the dependent variable using the same set of predictors, with eating habits score included as an explanatory variable. Standardized beta coefficients (*β*), unstandardized coefficients (*B*), 95% confidence intervals (CI), and model fit statistics (*R*^2^, adjusted *R*^2^, and *F*-statistics) were reported. Statistical significance was set at *p* < 0.05.

## Results

### Demographic characteristics of the study participants

The study participants included 343 female students with an age range of 18.5–44 years (22.77 ± 4.34 years old). The participants had a mean body weight of 55.74 ± 11.59 kg, height of 157.94 ± 5.43 cm, 59.5% students were considered to have normal weight, while 17.5% were underweight, 16.9% were overweight, and 6.1% were categorized as obese. Of all the participants, 83.4% were unmarried. In terms of academic levels, the highest percentage (32.7%) was in levels 7–8, followed by 28.3% in levels 3–4, 20.1% in levels 5–6, and 19.0% at master’s level. In terms of program enrolment, the BSc Food and Nutrition was the highest at 58%, followed by BSc Clinical Nutrition at 23%, MSc Food and Nutrition at 14.3%, and MSc Clinical Nutrition at 4.7% (Table 1).

**Table 1:** Demographic profile of the students included in the study (*n* = 343).

Variable	Mean ± SD	Range
Age (year)	22.77 ± 4.34	18.5–44
Weight (kg)	55.74 ± 11.59	32–98
Height (cm)	157.94 ± 5.43	146–176

Variable	Category	*n* (%)
BMI class	Underweight	60 (17.5%)
	Normal	204 (59.5%)
	Overweight	58 (16.9%)
	Obese	21 (6.1%)
Marital status	Unmarried	286 (83.4%)
	Married	57 (16.6%)
Academic level	Level 3–4	97 (28.3%)
	Level 5–6	69 (20.1%)
	Level 7–8	112 (32.7%)
	Master	65 (19.0%)
Types of programs	BSc Department of Clinical Nutrition, Faculty of Applied Medical Sciences	79 (23%)
	BSc Food and Nutrition Department, Faculty of Human Sciences and Design	199 (58%)
	MSc of Science in Clinical Nutrition, Faculty of Applied Medical Sciences	16 (4.7%)
	MSc Nutrition Sciences Program, Food and Nutrition Department, Faculty of Human Sciences and Design	49 (14.3%)

For the continuous variables, data are presented as mean ± SD and range. For the categorical variables, data are presented as *n* (%).

### Students’ knowledge and awareness of their nutritional requirements and healthy diet

As shown in Table 2, most participants demonstrated awareness of key healthy eating concepts. Specifically, 95.3% reported that a healthy diet promotes health and prevents disease, 90.4% recognized that a healthy diet provides the nutrients required by the body, 81.0% indicated that a healthy diet avoids substances harmful to health, and 81.6% reported knowing their daily nutritional requirements. These findings suggest that awareness of fundamental healthy eating principles was common among the study participants.

**Table 2:** Knowledge and awareness of self-nutritional requirements and healthy diet (*n* = 343).

Variable	Response	*n* (%)
A healthy diet promotes health and prevents diseases	No	16 (4.7%)
	Yes	327 (95.3%)
A healthy diet provides sufficient nutrients the body needs	No	33 (9.6%)
	Yes	310 (90.4%)
A healthy diet avoids consuming substances that are harmful to health	No	65 (19%)
	Yes	278 (81%)
Knowledge of daily self-nutritional requirements	No	63 (18.4%)
	Yes	280 (81.6%)

Data are presented as *n* (%).

### The pattern of eating habits among the students

As for the students who participated in the study, students had the higher proportion of eating snacks 2 times a day (39.7%), followed by 1 time a day (30.9%). When it came to healthier options, students most frequently described eating vegetables and fruits 1 time a day (38.2 and 35.0%, respectively). Eating habits for dates demonstrated a more varied pattern across the students, having almost equal proportions describing eating them 1 time a day (25.4%), rarely (23.9%), and 1–2 times a week (22.7%). In contrast, the students described eating fried food and fast food more frequently at the 1–2 times a week (35.3 and 39.1%, respectively). Eating a meal outside home (not at the university campus) was the most rarely described at 42.6%, while eating a meal at the university campus was most frequently described at 1–2 times a week (41.4%). Meals ordered through food delivery apps were most frequently described 1–2 times a week (33.8%), and rarely at 29.4%. Having a balanced diet in meals was found in 28% of them once per day and sharing food with friends reported in 24% of them 1–2 times per week while with family reported in 37.6% of them twice per day. All variables describing eating habits had a statistically significant difference in their distributions across the frequency categories (*p* < 0.001) (Table 3).

**Table 3:** Eating habits distribution among the participating students (*n* = 343).

Variable	Twice a day		Once a day		3–4 times a week		1–2 times a week		Rare	
	*n*	%	*n*	%	*n*	%	*n*	%	*n*	%
Eating snacks	136	39.7	106	30.9	58	16.9	31	9	12	3.5
Consuming vegetables	68	19.8	131	38.2	50	14.6	77	22.4	17	5
Consuming fruits	49	14.3	120	35	68	19.8	69	20.1	37	10.8
Consuming dates	37	10.8	87	25.4	78	22.7	59	17.2	82	23.9
Eating fried food	29	8.5	37	10.8	121	35.3	83	24.2	73	21.3
Eating fast food	24	7.0	36	10.5	134	39.1	63	18.4	86	25.1
Eating a meal outside the home	30	8.7	59	17.2	61	17.8	47	13.7	146	42.6
Eating a meal at the university campus	25	7.3	26	7.6	142	41.4	58	16.9	92	26.8
Eating a meal from food delivery applications	32	9.3	30	8.7	116	33.8	64	18.7	101	29.4
Having a balanced diet in meals	75	21.9	96	28	56	16.3	95	27.7	21	6.1
Sharing meals with friends	34	9.9	43	12.5	83	24.2	63	18.4	120	35
Sharing meals with family	129	37.6	95	27.7	32	9.3	77	22.4	10	2.9

### Dietary practices and their frequency among the students

The majority of students were able to eat meals regularly, with more than half doing so (54.2%) for sometimes and 30.0% for always. Eating breakfast daily was reported by 34.1% as always and 39.7% as sometimes, while breakfast was eaten outside home mainly (45.2%) as rarely and 33.2% as sometimes. Many students made healthier choices, with plant sources in the main meal either sometimes (42.3%) or always (29.4%) and in the same way considered the nutrient value sometimes (40.8%) or always (38.2%) and more than half of the students (36.4%) as always adding iodized salt during cooking. Staying on any healthy diet was most commonly reported as sometimes (39.4%), 17.8% reported for always, and 20.4% for never. All practices demonstrated significance in the response categories (*p* < 0.001) (Table 4).

**Table 4:** Frequency of dietary practices among the students (*n* = 343).

Variable	Always		Sometimes		Rare		Never	
	*n*	%	*n*	%	*n*	%	*n*	%
Eat meals regularly	103	30	186	54.2	44	12.8	10	2.9
Eat breakfast daily	117	34.1	136	39.7	83	24.2	7	2
Eat breakfast outside home	35	10.2	114	33.2	155	45.2	39	11.4
Consider a plant source in the main meal	101	29.4	145	42.3	72	21	25	7.3
Consider the nutrient value	131	38.2	140	40.8	58	16.9	14	4.1
Add iodized salt while cooking food	125	36.4	102	29.7	60	17.5	56	16.3
Follow a healthy diet	61	17.8	135	39.4	77	22.4	70	20.4

Data are presented as *n* (%). *p* values were calculated using the chi-square test.

### Frequency of reading food labelling and calories on the menu among the students

Students showed some degree of interest and involvement with the nutrition information provided. A total of 40.5% of students reported reading the nutrient composition label always, while 42.3% reported reading sometimes, and 4.1% of students reported never reading the nutrient composition label. Consideration of the health effects of foods based on the nutrition facts label was reported as always and sometimes by a similar percentage 37.9% of the students. Only 15.5% of the students always calculated the total calories consumed in a day. Also, 34.4% look at the calories on the menus when/before choosing order from restaurants sometimes, 36.2% sometimes the number of calories displayed on menus influenced their order and 40.5% reported that if the food delivery application provided them with information about calories on the menu, they would sometimes consider buying lower calorie foods. Significant differences were observed among all items queried (*p* < 0.001) (Table 5).

**Table 5:** Frequency of reading food labelling and calories on the menu among the students (*n* = 343).

Variable	Always		Sometimes		Rare		Never	
	*N*	%	*N*	%	*N*	%	*N*	%
Read the nutrients composition label	139	40.5	145	42.3	45	13.1	14	4.1
Consider the health effects of the food product based on the nutrition facts	130	37.9	130	37.9	66	19.2	17	5
Regularly calculate the daily consumed calories	53	15.5	101	29.4	100	29.2	89	25.9
Look at the calories on the menus when/before choosing an order from restaurants	102	29.7	118	34.4	78	22.7	45	13.1
The number of calories displayed on menus influenced the order	90	26.2	124	36.2	71	20.7	58	16.9
If the food delivery application provided you with information about calories on the menu, would you consider buying lower-calorie foods?	111	32.4	139	40.5	50	14.6	43	12.5

Data are presented as n (%). P values were calculated using the chi-square test.

### Barriers to healthy eating among students

Barriers to healthy eating among participants were summarized in Table 6, which include student, educational, food, social, social media, and food delivery application-related factors. Regarding student-related factors, most students did not think student-related issues were a large barrier to a healthy diet. The most disagreed with the statement was not interested in eating a healthy diet (60.6%). Concerning the educational factors, the majority of participants also disagreed that educational factors were a significant barrier, with 56.0% showing the highest disagreement on the item, insufficient knowledge/information about healthy diets. Food was also an important barrier. The key result was high agreement that fast foods are easily available and quickly accessible (67.3%), high cost of healthy foods (63.0%), and appealing promotion of unhealthy foods (55.1%). Environmental barriers included limited time to prepare healthy foods (52.8%). The social aspect was less prominent, as the majority of students disagreed that family does not promote healthy eating (42.9%) and peer influence promotes poor eating habits (42.6%). There was definitely a huge effect from social media. The most significant finding was the agreement by 63.6% of students that social media has an impact on the awareness of food decisions. Food delivery app-related factors were also most prevalent. The most prevalent finding was rewarding myself with new food, enjoying the food, and making memories (72.0%). Following it was ordering up at special times, for instance, exam period, stress, vacation, and boring period (62.4%). The main barriers appeared to be on food factors, social media, and food delivery apps.

**Table 6:** Barriers toward healthy eating among the participating students (*n* = 343).

Variable	Agree		Neutral		Disagree	
	*N*	%	*N*	%	*N*	%
Student factors						
Not interested in eating a healthy diet	30	8.7	105	30.6	208	60.6
No physical activity, exercise, or sport	56	16.3	119	34.7	168	49
Too lazy to prepare healthy foods	100	29.2	116	33.8	127	37
Non-periodical follow-up of nutritional status	102	29.7	130	37.9	111	32.4
An unhealthy diet is used as a coping strategy for stress	97	28.3	115	33.5	131	38.2
Educational factors						
Inadequate knowledge/information about healthy diets	67	19.5	84	24.5	192	56
Limited literature or books related to healthy eating	66	19.2	108	31.5	169	49.3
Unavailability of a healthy diet	68	19.8	93	27.1	182	53.1
Cooking healthy food is more difficult than cooking junk food	89	25.9	90	26.2	164	47.8
Limited variety of healthy foods	102	29.7	100	29.2	141	41.1
Good taste and food preferences for an unhealthy diet	91	26.5	134	39.1	118	34.4
Fast foods are easily and quickly available	231	67.3	77	22.4	35	10.2
Attractive advertising of an unhealthy diet	189	55.1	95	27.7	59	17.2
High cost of healthy foods	216	63	87	25.4	40	11.7
Environmental factors						
College atmosphere is not a suitable place to eat a healthy diet	143	41.7	98	28.6	102	29.7
Limited time to prepare healthy foods	181	52.8	103	30	59	17.2
Social factors						
Family is not supportive of an eating healthy diet	89	25.9	107	31.2	147	42.9
Pressure from peers to engage in unhealthy eating	89	25.9	108	31.5	146	42.6
Cultural and traditional food is not healthy	89	25.9	142	41.4	112	32.7
Social media factors						
Social media affects your awareness of your food choices.	218	63.6	86	21.5	39	11.4
The type of advertising (chocolate images, fast food videos, healthy food promotional content) affects your food purchases.	177	51.6	124	36.2	42	12.2
Do you purposefully follow people who publish publications about eating and food?	168	49	89	25.9	86	25.1
How much do influencer advertising sources with attractive colours and professional photos influence your food choices?	148	43.1	137	39.9	58	16.9
Ordering unhealthy food from food delivery applications increases						
After watching an advertisement and following food trends	153	44.6	116	33.8	74	21.6
During hedonic hunger time (craving for a specific unhealthy food)	204	59.5	91	26.5	48	14
During specific times (exam period and stress period, vacation, and feeling bored)	214	62.4	86	25.1	43	12.5
Rewarding myself (by experiencing new food, enjoying food, creating memories)	247	72	64	18.7	32	9.3
Eating out unhealthy food						
After watching an advertising and following food trends	159	46.4	107	31.2	77	22.4
During hedonic hunger time (craving for a specific unhealthy food)	189	55.1	101	29.4	53	15.5
During specific time (exams period and stress, period, vacation, feeling bored)	188	54.8	100	29.2	55	16
Rewarding myself (by experiencing new food, enjoying food, create memories)	247	72	64	18.7	32	9.3

### Correlation matrix between eating behaviors, practices and perceived barriers

A Pearson correlation analysis was conducted to examine the relationships among eating habits, dietary practices, food labelling and calorie menu use, and perceived barriers to healthy eating among female nutrition students. Eating habits were moderately and positively correlated with dietary practices (*r* = 0.470, *p* < 0.001) and food labelling and calorie menu use (*r* = 0.300, *p* < 0.001). Dietary practices were also strongly associated with food labelling behaviors (*r* = 0.540, *p* < 0.001), indicating that students with healthier dietary practices were more likely to use nutritional information when selecting foods.

Given that higher barrier scores reflect lower perceived barriers, positive correlations indicate that healthier behaviors were associated with fewer barriers. Eating habits were positively associated with student barriers (*r* = 0.357, *p* < 0.001), food barriers (*r* = 0.256, *p* < 0.001), social media barriers (*r* = 0.175, *p* < 0.01), ordering unhealthy food barriers (*r* = 0.146, *p* < 0.01), and eating-out unhealthy food barriers (*r* = 0.153, *p* < 0.01). Similarly, dietary practices were positively correlated with student barriers (*r* = 0.374, *p* < 0.001), food barriers (*r* = 0.256, *p* < 0.001), educational barriers (*r* = 0.125, *p* < 0.05), environmental barriers (*r* = 0.121, *p* < 0.05), social media barriers (*r* = 0.127, *p* < 0.05), ordering unhealthy food barriers (*r* = 0.182, *p* < 0.001), and eating-out unhealthy food barriers (*r* = 0.152, *p* < 0.01). Food labelling and calorie menu use were positively associated with student barriers (*r* = 0.334, *p* < 0.001), food barriers (*r* = 0.198, *p* < 0.001), and environmental barriers (*r* = 0.197, *p* < 0.001).

Among the barrier domains, significant positive intercorrelations were observed (*r* = 0.138–0.669, *p* < 0.05), suggesting that students reporting fewer barriers in one domain tended to report fewer barriers in others. The strongest associations were observed between ordering unhealthy food barriers and eating-out unhealthy food barriers (*r* = 0.669, *p* < 0.001), and between social media barriers and ordering unhealthy food barriers (*r* = 0.535, *p* < 0.001). Overall, healthier eating habits, healthier dietary practices, and greater use of nutritional information were associated with lower perceived barriers to healthy eating, particularly student-related and food-related barriers (Table 7).

**Table 7:** Correlation matrix between eating habits, dietary practices, and perceived barriers (*n* = 353).

Variable	Eating habits	Dietary practice	Food labelling and calories on menu	Student barriers	Educational barriers	Food barriers	Environmental barriers	Social barriers	Social media barriers	Ordering unhealthy food barriers^#^	Eating out unhealthy barriers
Eating habits	1.000										
Dietary practices	0.470***	1.000									
Food labelling and calories on menu	0.300***	0.540***	1.000								
Student barriers	0.357***	0.374***	0.334***	1.000							
Educational barriers	0.034	0.125*	0.097	0.264***	1.000						
Food barriers	0.256***	0.256***	0.198***	0.482***	0.447***	1.000					
Environmental barriers	0.086	0.121*	0.197***	0.248***	0.303***	0.452***	1.000				
Social barriers	0.074	0.080	0.064	0.272***	0.373***	0.438***	0.353***	1.000			
Social media barriers	0.175**	0.127*	−0.023	0.261***	0.192***	0.461***	0.290***	0.344***	1.000		
Ordering unhealthy food barriers^**#**^	0.146**	0.182***	0.062	0.307***	0.138*	0.465***	0.353***	0.243***	0.535***	1.000	
Eating out unhealthy barriers	0.153**	0.152**	0.005	0.212***	0.198***	0.430***	0.302***	0.270***	0.491***	0.669***	1.000

# Ordering unhealthy food from food delivery applications increases. *p < 0.05; **p < 0.01; ***p < 0.001.

### Multiple linear regression predicting eating habits and dietary practices

**Figure 1:**
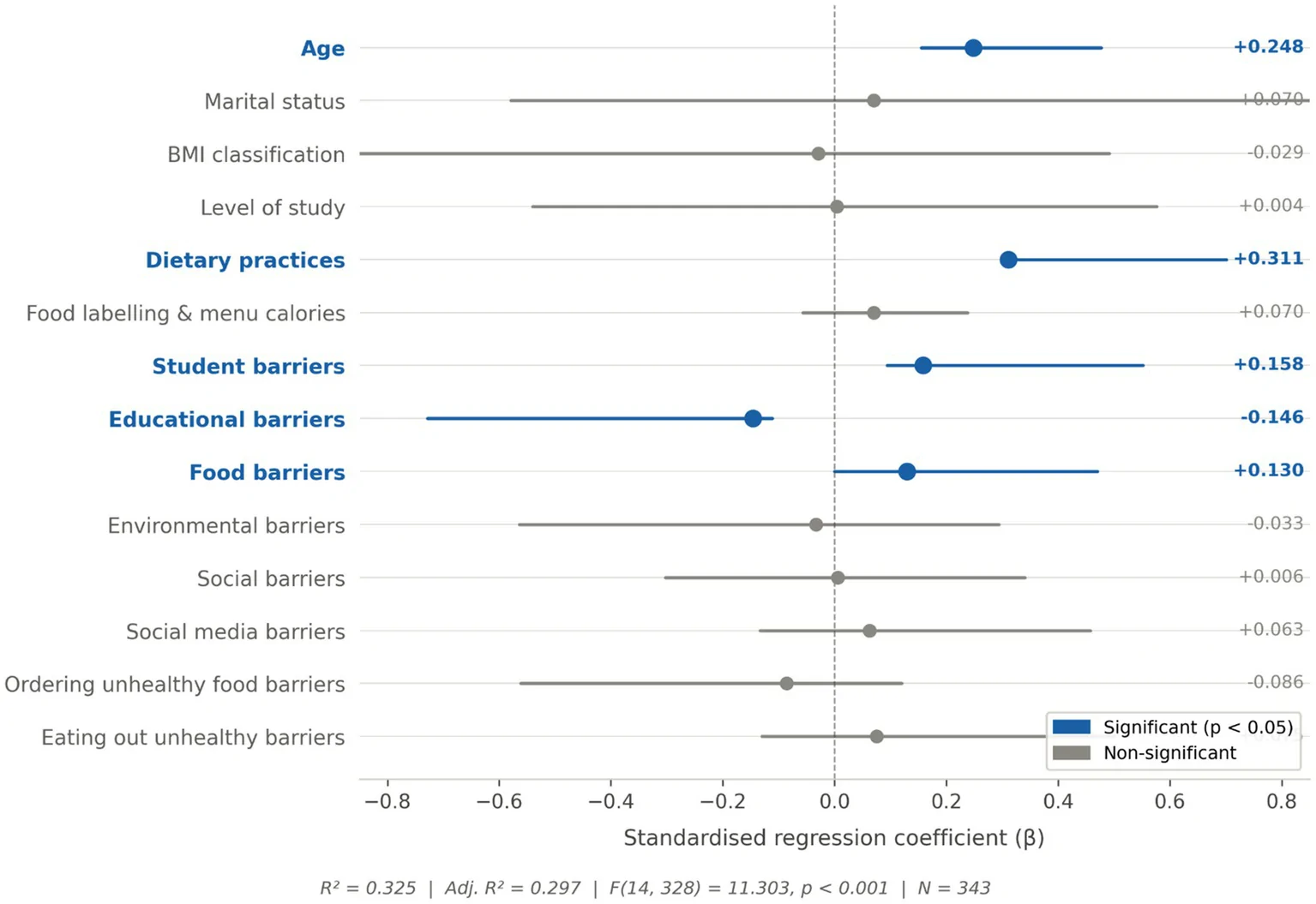
Multiple linear regression predicting eating habits with standardized coefficients (*β*) with 95% confidence intervals.

**Figure 2:**
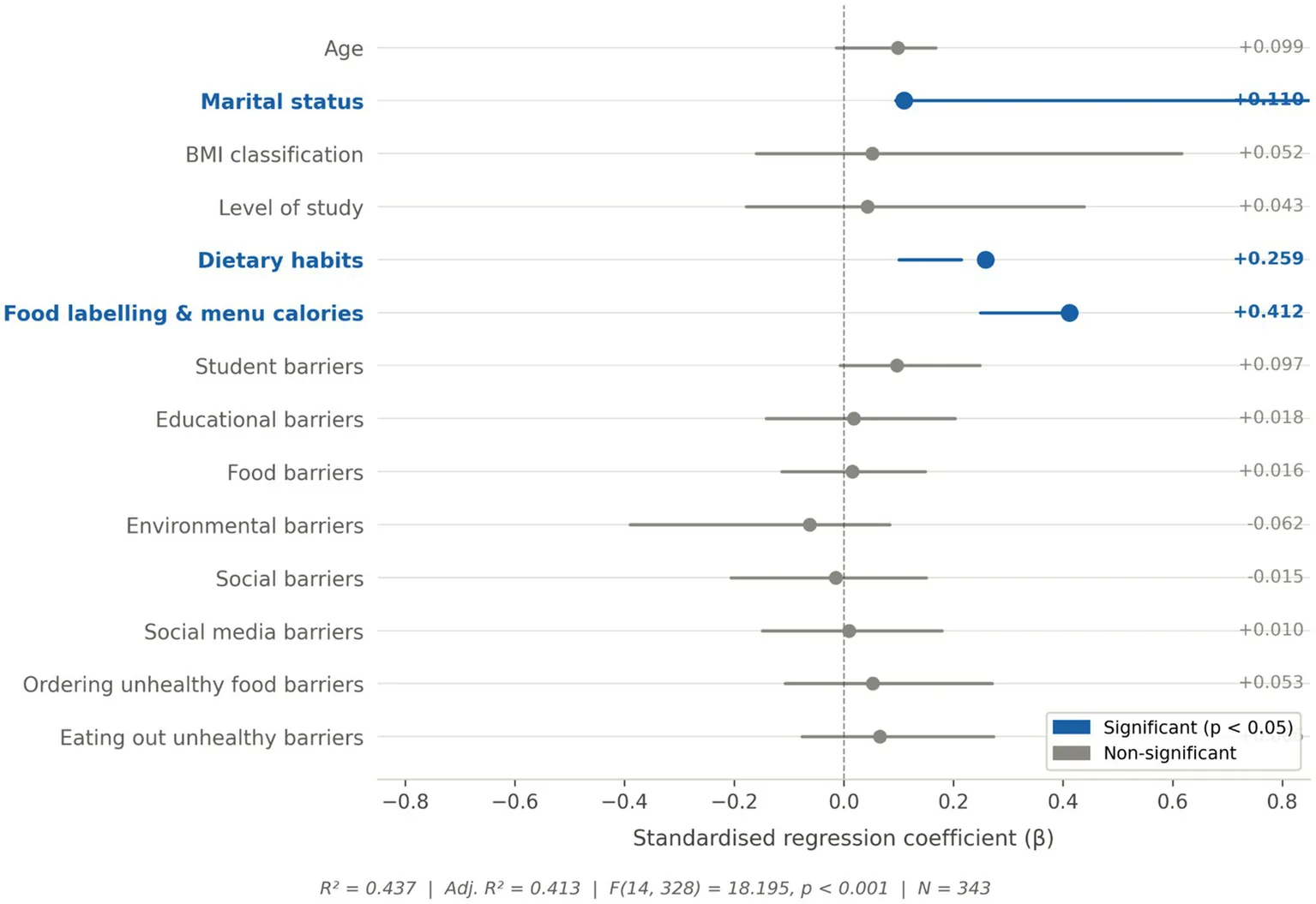
Multiple linear regression predicting dietary practices with standardized coefficients (*β*) with 95% confidence intervals.

A multiple linear regression analysis was conducted to identify predictors of eating habits (Figure 1) and dietary practices (Figure 2) among female nutrition students in Saudi Arabia. For eating habits, the overall model was statistically significant (*F*(14,328) = 11.303, *p* < 0.001), explaining 32.5% of the variance in eating habits (*R*^2^ = 0.325; Adjusted *R*^2^ = 0.297). Among the predictors, dietary practices emerged as the strongest positive predictor of healthier eating habits (*β* = 0.311, *p* < 0.001), indicating that students with healthier dietary practices also reported healthier eating habits. Age was also positively associated with eating habits (*β* = 0.248, *p* < 0.001), suggesting that older students tended to have healthier eating habits. In addition, student barriers were positively associated with eating habits (*β* = 0.158, *p* = 0.006). Given that higher barrier scores reflect lower perceived barriers, this finding indicates that students perceiving fewer student-related barriers reported healthier eating habits. Similarly, food barriers showed a positive association at the threshold of statistical significance (*β* = 0.130, *p* = 0.050), suggesting that fewer food-related barriers were associated with healthier eating habits. Conversely, educational barriers were negatively associated with eating habits (*β* = −0.146, *p* = 0.008). Because higher educational barrier scores indicate fewer perceived educational barriers, this result suggests that students reporting fewer educational barriers tended to have slightly poorer eating habits after adjustment for other variables. Marital status, BMI classification, level of study, food labelling and calorie menu use, environmental barriers, social barriers, social media barriers, ordering unhealthy food barriers, and eating-out unhealthy food barriers were not significant predictors of eating habits (all *p* > 0.05).

A multiple linear regression analysis was conducted to identify predictors of dietary practices among female nutrition students in Saudi Arabia (Figure 2). The overall model was statistically significant (*F*(14,328) = 18.195, *p* < 0.001), explaining 43.7% of the variance in dietary practices (*R*^2^ = 0.437; Adjusted *R*^2^ = 0.413).

Among the predictors, food labelling and calorie menu use was the strongest positive predictor of healthier dietary practices (*β* = 0.412, *p* < 0.001), indicating that students who more frequently used nutrition labels and calorie information reported healthier dietary practices. Eating habits were also positively associated with dietary practices (*β* = 0.259, *p* < 0.001), suggesting that healthier eating habits were linked to healthier dietary practices. In addition, marital status was a significant positive predictor (*β* = 0.110, *p* = 0.031), indicating differences in dietary practices according to marital status. Perceived barriers did not independently predict dietary practices after adjustment for other variables.

## Discussion

This study aimed to identify barriers to healthy eating and describe dietary practices among female university students enrolled in nutrition programs at King Abdulaziz University (KAU), Jeddah, Saudi Arabia. It revealed that the majority of undergraduate and postgraduate nutrition students believed that a healthy diet is beneficial for health, protects from disease, provides sufficient nutrients, and needs awareness of nutritional intake in daily life. Nevertheless, in the context of the reported dietary behavior and eating pattern, the general definition of the dietary pattern was only partially healthy. Measured against the WHO’s (31) benchmark of a balanced, diverse diet low in free sugars and energy-dense foods, the present sample fell short in concrete ways: nearly 40% of students snacked at least twice daily, more than a third consumed fried food and fast food 1–2 times a week, and fewer than one in five ate vegetables more than once a day. Accordingly, the current study does not demonstrate a pattern that can be described as healthy by this standard.

One of the most interesting results from this study is the existence of a significant knowledge–behavior gap. Despite nutrition students being the participants, who were expected to have better nutritional knowledge than the non-nutrition students, this knowledge had not been put into practice. The results of the present study suggest that participants had an inconsistent pattern of diet, regularly eating fruits and vegetables, while also regularly consuming snack, fried food, and fast food, which suggest overall diet lacked a healthy pattern, as healthy eating behaviors such as regular meals, regular breakfast, and temporary healthy food choices being reported as “sometimes” more frequently than “always.” This gap is notable precisely because the sample was not a general student population but students already trained in the WHO criteria described above; classroom knowledge of a healthy diet did not, by itself, translate into more frequent breakfast consumption, more regular meals, or less frequent snacking and fast-food intake. The moderate correlation between eating habits and dietary practices observed in this study (*r* = 0.470, *p* < 0.001) suggests that these behavioural domains move together, but neither is strongly anchored to the nutrition awareness or knowledge reported separately, suggesting knowledge functioned largely independently of day-to-day food choice in this sample. This outcome is consistent with earlier research evidence that nutrition knowledge is associated with higher diet quality, but the extent of the association is usually weak to modest and not strong (32–34). A comparable knowledge–behavior dissociation has also been reported among University students, where good nutritional knowledge coexisted with unhealthy eating habits (23, 35–37), suggesting this disconnect is a broader feature of university students.

Moreover, the results imply that dietary behavior in this population is determined not only by knowledge or awareness but also by the general university food environment and routine practical constraints. In this study, the most heavily endorsed barriers were practical and environmental rather than motivational: 67.3% of students agreed that fast food is easily and quickly available, 63.0% cited the high cost of healthy foods, and 52.8% reported limited time to prepare healthy meals compared with only 8.7% who cited simple disinterest in eating healthily. This ordering indicates that, for this population, the obstacle is less a matter of attitude than of an environment that makes the unhealthy option the cheaper, faster, and more accessible one. In literature, similar environmental and convenience-driven barriers have been described in qualitative studies, systematic reviews, and mixed-methods university investigations, where students’ food behaviors were dictated by taste, accessibility, affordability, convenience, campus habits, peer pressures, and chronic lack of time (1, 8, 38–40). Thus, the unsteady eating habits present in this study most likely reflect how dietary knowledge functions alongside a food environment that frequently provides fast, low-cost, and energy-dense products. The correlation and regression analyses that follow extend this descriptive picture by clarifying how eating habits, dietary practices, food-labelling behavior, and perceived barriers relate to, and predict, one another.

A Pearson correlation analysis clarified these relationships statistically. Eating habits, dietary practices, and food-labelling and calorie-menu use were all positively and significantly intercorrelated (*r* = 0.300–0.540), with the strongest association between dietary practices and food-labelling behavior (*r* = 0.540, *p* < 0.001). This pattern suggests the three behavioural domains are not independent but reflect a broader orientation toward nutrition-conscious self-regulation, consistent with evidence that nutrition knowledge and label literacy translate, at least partially, into healthier intake (32, 33). Healthier behaviors were also associated with fewer perceived barriers, particularly within student-related and food-related domains (*r* = 0.256–0.374, *p* < 0.001), while associations with educational, environmental, and social barriers were weaker or non-significant, reinforcing the descriptive finding above that food- and convenience-related obstacles, rather than knowledge gaps per se, are most proximally linked to actual eating behavior in this sample (8, 38–40), and mirroring findings from King Faisal University, where practical, food-related barriers had a stronger bearing on dietary adherence than informational ones among Saudi students (23). The barrier domains were also substantially intercorrelated with one another (*r* = 0.138–0.669), most strongly between food-delivery-ordering and eating-out barriers (*r* = 0.669, *p* < 0.001), indicating that barriers cluster together rather than operate as isolated, independent obstacles consistent with ecological models that frame individual, social, and environmental influences on student eating behavior as interacting layers (41).

Building on the strong correlation between dietary practices and food-labelling behavior noted above, findings regarding food-label reading and menu calorie use were more encouraging than the eating-habits data alone would suggest: 40.5% of students reported always reading nutrient composition labels and 82.8% did so at least sometimes, yet only 15.5% always calculated their total daily calorie intake. This gap between passive label-checking and active calorie tracking points to a medium level of nutrition-related self-regulation students engage with available information but stop short of using it to actively quantify intake. This is consistent with the regression finding that food-labelling and calorie-menu use was the strongest independent predictor of healthier dietary practices in this sample (*β* = 0.412, *p* < 0.001)—stronger than any barrier variable indicating that, of all factors measured, label-related behavior carried the most explanatory weight for actual dietary practice. Such engagement with nutrition information aligns with evidence that nutrition labels have a modest positive impact on dietary intake among college students (42, 43). Moreover, an experiment using a virtual food-delivery app proved that calorie labelling is effective in order to reduce calories in some of the outlets (44), which relevant given that food-delivery ordering was itself one of the most commonly endorsed barriers in this study (72.0% reward-related ordering). The current findings, however, raise new questions about whether labelling may promote better choice, as information alone may not be enough.

A further insight from the current study is that emotional, situational, and social cueing appears to influence unhealthy purchases. Participants identified self-reward (72.0%) and specific stress-related periods—exams, vacations, boredom (62.4%)—and hedonic hunger or craving (59.5%) as the leading reasons for ordering unhealthy food through delivery apps, all of which outranked simple advertising exposure (44.6%) as a stated driver. This ranking suggests that, for this sample, unhealthy ordering functions more as an emotion-regulation or coping behavior than as a response to marketing per se, implying that messaging targeting advertising exposure may have less impact than strategies offering alternative coping mechanisms during highstress periods such as exams. Similar findings occurred regarding how undergraduate students with high perceived stress consumed more health-ineffective dietary behaviors, such as shopping more often for convenience foods (45). This pattern is broadly consistent with ecological models of student eating behavior, in which time pressure, academic stress, and social context interact to undermine otherwise healthy intentions (1, 38, 41). This clustering of pressures is consistent with the broader barrier intercorrelations described above (up to *r* = 0.669 between food-delivery-ordering and eating-out barriers), reinforcing that these are not isolated obstacles but interrelated ones. Social media exposure followed a similar logic here: 63.6% of students agreed that social media affects their awareness of food choices, and this barrier correlated significantly with food-related (*r* = 0.461, *p* < 0.001) and food-delivery-ordering barriers (*r* = 0.535, *p* < 0.001), indicating that social-media influence and situational ordering behavior cluster together rather than acting as separate pressures. Furthermore, the current research findings on advertising and media influence are consistent with other studies that have found social media use influences food selection in college-aged people and that heavy social media use may be detrimental to college students’ nutritional intake (14, 46).

These correlational patterns were further clarified by multiple linear regression analyses identifying which factors retained independent explanatory value once others were accounted for. For eating habits, the overall model was statistically significant (*F*(14,328) = 11.303, *p* < 0.001) and explained 32.5% of the variance (*R*^2^ = 0.325). Dietary practices were the strongest predictor (*β* = 0.311, *p* < 0.001), reinforcing the bivariate correlation reported above and suggesting the two behavioural domains are mutually reinforcing rather than driven by entirely separate determinants. Age was also an independent positive predictor (*β* = 0.248, *p* < 0.001), broadly compatible with the view that university is a transitional period during which dietary self-regulation may improve somewhat as students gain independence and progress academically (4). Student-related barriers remained a modest independent predictor (*β* = 0.158, *p* = 0.006), but food-related barriers fell to borderline significance (*β* = 0.130, *p* = 0.050) once dietary practices, label use, and demographic variables were entered simultaneously, representing a marked change from their stronger bivariate association noted above. The negative coefficient observed for educational barriers (*β* = −0.146, *p* = 0.008) is counterintuitive given their positive bivariate correlation, and most plausibly reflects statistical suppression arising from the substantial intercorrelation among barrier domains (up to *r* = 0.669) rather than a genuine adverse effect of fewer educational obstacles; this coefficient should be interpreted with caution rather than at face value.

A comparable pattern emerged for dietary practices (*F*(14,328) = 18.195, *p* < 0.001; *R*^2^ = 0.437): food-labelling and calorie-menu use was the strongest independent predictor (*β* = 0.412, *p* < 0.001, consistent with the descriptive finding above), followed by eating habits (*β* = 0.259, *p* < 0.001) and, more modestly, marital status (*β* = 0.110, *p* = 0.031). Notably, none of the perceived-barrier domains remained significant predictors of dietary practices after adjustment, despite several showing significant bivariate correlations. Taken together, the two regression models suggest that perceived barriers may influence dietary behavior largely indirectly through their association with nutrition-related skills such as label use and existing habits rather than exerting strong independent effects once these behavioural competencies are accounted for. This is consistent with prior work in nutrition- and dietetic-student populations showing that academic training and barrier awareness do not, by themselves, reliably translate into improved dietary behavior without parallel gains in practical, self-regulatory skills (17, 18, 47), and with reviews concluding that knowledge is a necessary but insufficient driver of healthier eating among university students (34). As these associations derive from a cross-sectional design, causal direction cannot be established. It is equally plausible that healthier behaviors reduce the perception of barriers as that fewer barriers facilitate healthier behavior. This caveat that should temper interpretation of the correlation and regression findings reported here.

### Practical implications

Findings of the present study indicate that barriers to healthy eating are not uniformly influential among female nutrition students at KAU. Food-related, student-related, and food delivery and social media-related barriers showed the strongest associations with dietary behavior. However, dietary practices themselves are most consistently and independently linked to nutrition-related skills (label use) and existing habits rather than to barrier removal alone. This is especially relevant considering the study population was future nutrition professionals for whom healthy eating is relevant not only on a personal level but also on a professional level. Similar limitations have been identified in research on dietetic-student populations in which taught nutrition was unable to overcome the practical, social, and structural barriers to healthy eating experienced during university life (47). The implications of the current study support training and curricular approaches that build practical, skill-based behaviors, such as habitual label use and meal planning. These interventions should pair nutrition knowledge delivery with barrier-reduction strategies that improve the affordability and availability of healthy food on campus, rather than assuming that nutrition awareness through the academic knowledge, in and of itself, would be sufficient to rule out the effects of convenience, cost, stress, media influence, and the on-campus food environment (48).

### Strengths and limitations

This study has several notable strengths. The questionnaire used had documented face validity, established through expert review, and acceptable-to-good internal consistency across all sections (Cronbach’s *α* = 0.71–0.78), supporting the reliability of the eating habits, dietary-practices, food-labelling, and barrier measures (27–30). By focusing specifically on female students already enrolled in nutrition programs, the study also addresses a population whose own dietary behavior carries particular professional relevance, as these students are future dietitians and nutrition professionals; their personal eating habits and the credibility this lends to patient counseling are directly tied to their future effectiveness as role models and educators for the populations they will serve. Finally, rather than relying on descriptive percentages alone, the dual-level analytic approach illustrated by the marked attenuation of several barrier domains once dietary practices and food-labelling behavior were entered into the regression models provides a more statistically robust and nuanced picture of the determinants of dietary practice in this population than descriptive statistics alone would allow.

In terms of limitations, the study was conducted among female students enrolled in nutrition programs at a single university, which may limit the generalizability of the findings to students from other universities, academic disciplines, regions, or to male students. In addition, detailed socioeconomic and living condition variables, such as household income, financial status, accommodation type, and residence arrangements, were not assessed. These factors may influence dietary behaviors and access to healthy foods and should be considered in future research. In addition, all dietary and behavioural data were collected through a self-administered, self-reported questionnaire, a method susceptible to social desirability bias and recall error, particularly for less socially desirable behaviors such as snacking, fried-food, or fast-food consumption (49).

## Conclusion

Students enrolling nutrition programs at KAU were aware of healthy-diet principles, but this awareness was only partly reflected in their dietary behaviors, which were moderately healthy overall. Practical and environmental barriers, particularly fast-food availability, cost, time constraints, and food-delivery-related pressures, showed the strongest associations with dietary behaviors, whereas educational and social barriers were less influential. Most barrier domains lost independent predictive value after accounting for eating habits and food-labelling behaviors, suggesting that barriers influence dietary behavior mainly through their effects on everyday habits and nutrition-related skills. Food-labelling and calorie-menu use was the strongest predictor of healthier dietary practices, while eating habits and dietary practices were positively linked. These findings suggest that knowledge or awareness alone is insufficient to ensure healthy eating, as environmental and lifestyle factors continue to shape food choices among future nutrition professionals.

### Recommendations

Interventions should move beyond knowledge-based education and focus on practical skills, particularly nutrition-label use, calorie awareness, and meal planning. Universities should improve the affordability, availability, and visibility of healthy food options and provide convenient, low-cost meal solutions. Programs should also address stress-related and emotional eating through coping strategies and support resources, while incorporating social media literacy to address food-delivery and eating-out influences. Because barriers appear to affect dietary practices mainly through habits and label use, interventions should combine environmental improvements with behavior- and skill-building strategies. Future studies should include more diverse student populations and use longitudinal or intervention designs to clarify causal relationships between perceived barriers and dietary behaviors.

## Data Availability

The original contributions presented in the study are included in the article/supplementary material, further inquiries can be directed to the corresponding author.
